# Dissemination and Implementation of Evidence Based Best Practice Across the High Value Healthcare Collaborative (HVHC) Using Sepsis as a Prototype – Rapidly Learning from Others

**DOI:** 10.5334/egems.192

**Published:** 2017-12-15

**Authors:** Andreas Taenzer, Allison Kinslow, Christine Gorman, Shelley Schoepflin Sanders, Shilpa J Patel, Sally Kraft, Lucy Savitz

**Affiliations:** 1 Dartmouth-Hitchcock, US; 2 High Value Healthcare Collaborative, US; 3 The Dartmouth Institute, US; 4 Providence St. Vincent Medical Center, US; 5 Hawaii Pacific Health, US; 6 Kaiser Permanente Center for Health Research, US

**Keywords:** Comparative Effectiveness Research, Electronic Health Records, Delivery of Health Care, Quality Improvement, Data Collection

## Abstract

The dissemination of evidence-based best practice through the entire health care system remains an elusive goal, despite public pressure and regulatory guidance. Many patients do not receive the same quality of care at different hospitals across the same health care system. We describe the role of a data driven learning collaborative, the High Value Healthcare Collaborative (HVHC), in the dissemination of best practice using adherence to the 3-hour-bundle for sepsis care. Compliance with and adoption of sepsis bundle care elements comparing sites with mature vs non-mature care delivery processes were measured during the improvement effort for a cohort of 20,758 patients. Non-mature sites increased their bundle compliance from 71.0 to 86.7 percent (p < 0.005). This compliance increase was primarily based on increased compliance with the fluid element of the bundle that improved for non-mature locations from 76.4 to 94.0 percent (p < 0.005).

## Introduction

The dissemination of evidence-based best practice through the entire health care system remains an elusive goal, despite public pressure and regulatory guidance. Many patients do not receive the same quality of care at different hospitals across the same health care system. The slow pace of adoption may be due to the absence of best practice or quality outcomes data in some settings [1]. The Institute for Healthcare Improvement (IHI) has promoted treatment bundles as a tool to bring evidence-based best practice to the bedside. IHI defines a bundle as “a structured way of improving the processes of care and patient outcomes: a small, straightforward set of practices—generally three to five—that, when performed collectively and reliably, have been proven to improve patient outcomes” [2].

The Surviving Sepsis Campaign (SSC) promoted bundles for the care of patients with sepsis [3] and these bundles were largely adopted by the Center for Medicare Services (CMS) as an all-or-none pay for performance (SEP-1). The 3-hour-bundle for sepsis consists of four elements: (1) drawing of a blood sample to determine lactate, (2) blood cultures prior to administering antibiotics, (3) providing broad spectrum antibiotics and (4) giving an intravenous fluid bolus of 30 ml kg^–1^. The increase of bundle compliance from 4.9 to 73.4 percent over a seven year time span was associated with a statistically significant decline in mortality from 21.7 percent in 2004 to 9.7 percent in 2010 [4]. In response to new evidence and CMS incentives, health systems across the nation are actively engaged in improving care for patients with sepsis.

The High Value Healthcare Collaborative (HVHC) is a consortium of health care delivery systems formed to improve quality of care while reducing cost [5]. In 2015, HVHC received an award from the Laura and John Arnold Foundation to test collaboration and training strategies by identifying and deploying the critical resources needed to support frontline health care teams as they learn how to provide the best care to patients with sepsis [6]. HVHC (http://highvaluehealthcare.org) is a unique quality improvement model which combines the features of a learning platform with sophisticated data collection and validation (described elsewhere in this journal), thereby providing a perfect environment in which to test whether such collaboratives promote rapid dissemination of best practices to improve adherence to the Surviving Sepsis 3-hour-bundle across a variety of health care systems. We refer the interested reader to other articles in this journal describing the creation of a learning platform, central data collection and verification, scheduled check-in calls, semi-annual in-person meetings, and site visits along with other tools. In this manuscript, we describe the changes of 3-hour-bundle compliance at sites self-identified as not having mature sepsis care delivery processes in place as compared to sites with mature sepsis care delivery systems.

## Methods

### Participating Centers

Eligible participants were members of the HVHC. The collaborative consists of five founding members (Dartmouth Hitchcock, Denver Health, Intermountain Healthcare, Mayo Clinic and The Dartmouth Institute) and eight collaborative members (Baylor Scott & White Health, Beth Israel Deaconess Medical Center, Hawai’i Pacific Health, Northwell Health, Providence St. Joseph’s Health, Sentara Healthcare, UC San Diego Health System, Virginia Mason Medical Center). All health care systems participated, except for one that was not a member at the beginning of the project. Dissemination of best practice was open to emergency departments, intensive care units and general care units. Because most sepsis patients initially present through emergency departments (ED), participation from EDs was greater than other units, and only data from the ED patient cohort is presented here.

Prior to engaging in the improvement effort of dissemination of evidenced-based best practice for sepsis patients, sites categorized themselves into either having a mature sepsis care process or not (called “mature” vs. “non-mature” subsequently); this was self-defined based on the implementation status of a sepsis care model. Sites that had planned, but not piloted or implemented a sepsis care process fell under the “non-mature” category while sites that had either piloted or implemented a standard model of sepsis care and were monitoring performance were in the “mature” category.

### Patient Cohort

Patients admitted with severe sepsis and septic shock via the emergency department from 10 participating HVHC sites in 2015 and 2016 were reviewed. Criteria for sepsis had to be present on arrival in the ED or be met in the ED. To be included, patients had to have known or suspected infection and either a lactate level of greater or equal to 2.0 mmol L^–1^, or a systolic blood pressure of less than 90 mm Hg, or a drop in consecutive systolic blood pressure readings of greater or equal to 40 mm Hg. Diagnosis of sepsis was made based on local processes and time zero was established following the Center for Medicare Services (CMS) SEP-1 guidelines [7]. Inclusion criteria were patients who had a discharge diagnosis (ICD 9/10) of severe sepsis or septic shock (according to CMS SEP-1 definition) and age over 18 years. Patients who received comfort measures only or had advanced directives that prevented the delivery of the 3-hour-bundle elements for sepsis were excluded, as the primary measure was compliance with the bundle. Other exclusion criteria based on SEP-1 or expert consensus were: Transfer from another acute care facility, length of stay (discharge date minus admission date) greater than 120 days, patients with sepsis who expired within 3 hours of presentation and patients receiving IV antibiotics for more than 24 hours prior to presentation.

### Care Bundle

HVHC adopted the Surviving Sepsis Campaign’s 3-hour-bundle for sepsis care [3] as there is broad support for its use, in contrast to subsequent interventions [8,9,10,11]. This bundle had been used at all HVHC sites who declared themselves as mature, hence the dissemination and implementation of a commonly used and evidence supported intervention such as the 3-hour-bundle found easy consensus among participants.

### Data Collection

Sites could choose between different levels of participation in regard to data submission: either uploading compliance with the 3 hour bundle in the form of “yes” or “no” flags, or contributing time stamped data for the process measures along with presenting lactate and blood pressure values. HVHC members uploaded data based on data specifications to a central secure server where data was validated (please see “A Framework for Aligning Data from Multiple Institutions to Conduct Meaningful Analytics” in this journal for details) for correct formatting as well as content. Once passing the quality checks, the data was analyzed and displayed on a member accessible website for review.

### Measures

**Figure 1:**
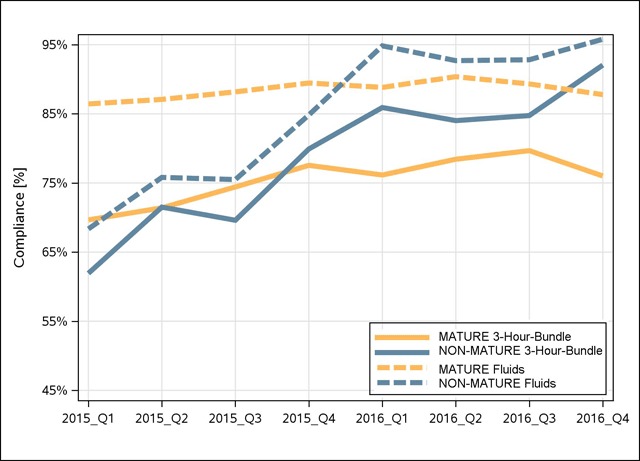
3-hour-bundle (Bundle) and intravenous fluid bolus (Fluids) compliance for Mature and Non-Mature sites from quarter one 2015 through quarter four of 2016 in percent. Non-Mature sites rapidly improved to match Mature sites.

The primary outcome measure was change in 3-hour-bundle compliance along with changes in the bundle elements between 2015 and 2016. Patients with a lactate of 2.0–3.9 mmol L^–1^ and normal blood pressure were part of this cohort that are not subject to the fluid bolus element of the 3-hour-bundle based on existing guidelines. In order to display all patients as one cohort, these patients were categorized as having met the 3-hour-bundle requirement. Hence, it is possible (as seen in Figure 1), that the 3-hour bundle compliance is greater than the compliance with administering the fluid bolus.

### Data Analysis

Results are shown in percentage or average +/– standard deviation (SD). Data was analyzed using Pearson’s Chi-squared test with Yates’ continuity correction and is graphically displayed as time series data. Analysis was done with R 3.3.2 [12] on Linux and figures were generated in Python 3.6 using the matplotlib [13] library. Though p-values are displayed for interest, data is not adjusted for factors such comorbidity index, age, gender, or other possible confounders.

## Results

Five emergency departments were in the non-mature and 39 in the mature category. A total of 20,758 patients were part of the cohort, with 19,037 in the mature and 1,721 in the non-mature groups.

The average age for the mature site cohort was 68.8 years (+/–17.5) in 2015 and 66.2 years (+/–17.7) in 2016. Non-mature cohort was 70.4 years (+/–16.7) in 2015 and 69.6 years (+/–16.9) in 2016. Gender distribution was 46.6 percent and 46.4 percent female for mature; 47.8 percent and 43.8 percent for non-mature sites respectively (Table 1).

**Table 1:** Demographic data and In-Hospital Mortality for Mature and Not Mature sites.

	Mature		Not Mature	

	2015	2016	2015	2016

N	9236	9716	852	869
Age (avg, +/–SD)	68.8+/–17.5	66.2+/–17.7	70.4+/–16.7	69.6+/–16.9
Gender (F%)	46.6%	46.4%	47.8%	43.8%
In-Hospital Mortality (%, N)	15.1+/–0.36 N = 1,404	14.3+/–0.35 N = 1,385	18.9+/–0.39 N = 161	17.4+/–0.38 N = 151

In-hospital mortality was 15.1 percent (1,404 of 9,326 patients) in 2015 and 14.3 percent (1,385 of 9,716) in 2016 for mature site emergency department patients with severe sepsis and septic shock. The corresponding rates for non-mature sites were 18.9 percent (161 of 852) and 17.4 percent (151 of 869). These changes were statistically not significant.

Mature sites increased their 3-hour-bundle compliance from 73.7 to 77.6 percent (p < 0.005) while non-mature sites improved from 71.0 to 86.7 percent (p < 0.005). The compliance with the intravascular fluid bolus of 30 ml kg^–1^ increased for non-mature locations from 76.4 to 94.0 percent (p < 0.005), largely contributing to their 3-hour-bundle adherence improvement. Mature sites maintained their fluid bundle adherence, 87.9 percent for 2015 and 89.1 percent for 2016 (Table 2 and Figure 1).

**Table 2:** 3-hour-bundle and intravenous fluid bolus change between 2015 and 2016 for mature and not mature sites. NS = not significant, * p < 0.005.

	Mature		Not Mature	

	2015 (N = 9326)	2016 (N = 9716)	2015 (N = 852)	2016 (N = 869)

Fluid Bolus [%]	87.9+/–32.6	89.1+/–31.1	76.4+/–42.5	94.0+/–23.7
	+1.2% (NS)		+19.6%*	
3-hr-Bundle [%]	73.7+/–44.0	77.6+/–41.7	71.0+/–45.4	86.7+/–34.0
	+3.9%*		+15.3%*	

## Discussion

**Figure 2:**
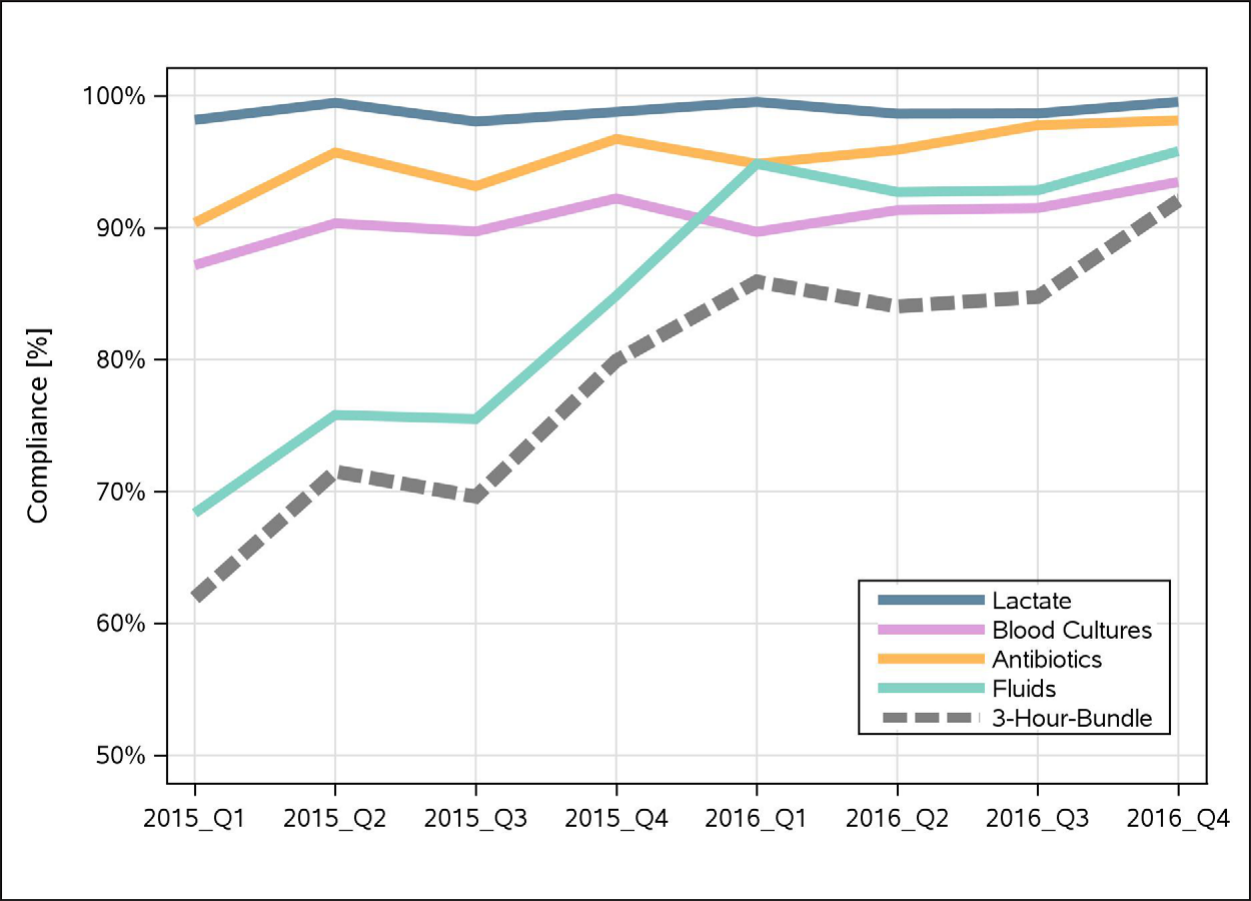
3-hour-bundle compliance and bundle elements over time for Non-Mature emergency departments demonstrating that the increase in fluid compliance was responsible for the improved 3-hour-bundle compliance.

Our results indicate that the dissemination of evidence-based best practice in learning collaboratives can lead to rapid improvement in care delivery in the case of sepsis. Non-mature sites improved their 3-hour-bundle compliance primarily by improving their adherence to the recommended intravenous fluid bolus (Figure 2), while the other bundle elements remained stable as did all for mature sites. This improvement in fluid adherence was no small challenge for the non-mature sites – the learning collaborative provided a forum to share resources, tools (such as quarterly “pulse checks” to front line providers and managers to assess facilitators and barriers to bundle implementation), data, solutions to common problems, and a mentored implementation model of monthly calls to troubleshoot, share successes and challenges, ask questions and provide a “sepsis community.”

The adoption of implementation sciences into health care improvement efforts is rapidly progressing. Four key variables have been described to impact the adoption of innovations in health care: 1) the external environment (e.g., external payors, such as CMS SEP-1 for sepsis care), 2) the organization of the health care delivery system, 3) the features of the change to be implemented (e.g., evidence for the 3-hour-bundle) and lastly, 4) the implementation process itself (e.g., local vs. part of an improvement collaborative) [14,15]. While these variables determine the success of dissemination and implementation, not all of them can be changed easily (the organization of the health care system).

For our project, the alignment with the anticipated CMS SEP-1 measure and associated financial incentives provided impetus for adoption, while the collaborative provided the structure within which to successfully disseminate the improvements and measure successes. The 3-hour-bundle is recommended by professional organizations, such as the Surviving Sepsis Campaign and the Society of Critical Care Medicine, as other external environment drivers.

While the evidence for the bundle is reasonable, it is not without discussion. The level of evidence for broad spectrum antibiotics is moderate but remains low for the fluid bolus [16]. It is the fluid bolus that generates the most discussion [17,18], and it is therefore not surprising that this bundle element had the lowest adoption among non-mature sites. Compliance with intravenous fluids has been the topic of multiple learning and educational sessions as part of this project (described elsewhere in this journal). The mentored implementation model promoted the sense of accountability for dissemination and implementation of best evidence according to our members. At the beginning of the project, members were asked to set targets for bundle compliance, percentage of patients discharged to home, length of stay and mortality. These target setting goals were shared among members and created a sense of accountability, that was fostered by pulse checks (monthly check in calls with review of performance data, barriers and successes). This environment of a support and accountability may have contributed to the rapid improvement among non-mature sites in fluid bolus delivery and overall 3-hour-bundle compliance.

### Limitations

The results of this project of accelerated adoption and implementation of evidenced-based best practice in sepsis care in a provider learning network committed to improving health care value through data and collaboration are intriguing. Nevertheless, there are numerous limitations to our findings. Members did not submit patient comorbidities, and while very unlikely, a significant change of comorbidities from one year to another (e.g., many more sepsis patients presenting with concurrent heart failure at baseline 2015 vs. 2016) may have influenced the compliance with fluid administration. Given the number of patients in this cohort, this is unlikely to be the case. Furthermore, mortality data is presented for informational purposes only. Mortality is not risk adjusted, and even though increased bundle compliance has been associated with decreased mortality [4,19], in the absence of adjustment we did not test for significance of change.

Could individual health care systems improve on their own at the same pace and with the same results? Generating evidence in implementation science and for complex changes in care delivery is difficult as many confounding factors cannot be accounted for. Bundle compliance among HVHC partners far exceed that of published results [20], indicating the high quality of care at the participant sites. Many members of the HVHC feel that they have benefited tremendously from shared learnings, joint data collection and review. We have seen rapid and accelerated dissemination and adoption of best practices across HVHC, in many cases shortening the 7 year timespan of improvement as described by Miller [4] in just a few months.

## Conclusion

The effect of quality improvement collaboratives on actual patient outcomes is difficult to estimate. Schouten et al. state in a systematic review of 72 publications on the impact of improvement collaboratives on patient outcomes: “Considering that quality improvement collaboratives seem to play a key part in current strategies focused on accelerating improvement, but may have only modest effects on outcomes at best, further knowledge of the basic components effectiveness, cost effectiveness, and success factors is crucial to determine the value of quality improvement collaboratives” [21].

In this journal we present in detail the components of a successful collaborative and in this manuscript, much improved process measures over a very short period of time, an impressive increase in compliance with evidence based best practice.
